# The Sendai framework: disaster risk reduction through a health lens

**DOI:** 10.2471/BLT.15.157362

**Published:** 2015-06-01

**Authors:** Amina Aitsi-Selmi, Virginia Murray

**Affiliations:** a Public Health England, 133-155 Waterloo Road, London, SE1 8UG, England.; b UNISDR Scientific & Technical Advisory Group, Geneva, Switzerland.

After negotiations at the World Conference on Disaster Risk Reduction in March 2015, a new framework was adopted by 187 Member States.^1^ The* Sendai framework for disaster risk reduction 2015–2030* highlights concerns on human health and well-being that are common to disaster risk reduction, climate change and sustainable development.^2^ A substantial emphasis on health is a welcome development, given the relative lack of attention to health issues in its predecessor, the *Hyogo framework for action 2005–2015*.^3^ The Hyogo framework did succeed in galvanizing many stakeholders including governments, scientists, the commercial sector and nongovernmental organizations to make progress on disaster risk reduction.^1^^,^^4^ However, more progress is needed in addressing underlying vulnerability from poverty, inequity or poor urban planning and land use.^5^ Disaster impacts are strongly influenced by physical, social, economic and environmental factors.^6^ Reducing disaster risk, therefore, requires concerted action across a wide range of sectors, institutions and disciplines. The Sendai framework is relevant within and beyond the health sector. There are more than 30 explicit references to health, referring to the implementation of an all-hazards approach to managing disaster risk, including links to epidemics and pandemics, several references to the *International health regulations* *(2005)*^7^ and to rehabilitation as part of disaster recovery.

Via the framework, Member States have called for enhanced scientific and technical work on disaster risk reduction and its mobilization through the coordination of existing networks and scientific research institutions at all levels and all regions, with the support of the United Nations International Strategy for Disaster Reduction’s Scientific and Technical Advisory Group, in order to strengthen the evidence base in support of the implementation of this framework; promote scientific research of disaster risk patterns, causes and effects; disseminate risk information with the best use of geospatial information technology; provide guidance on methodologies and standards for risk assessments, disaster risk modelling and the use of data; identify research and technology gaps and set recommendations for research priority areas in disaster risk reduction; promote and support the availability and application of science and technology to decision-making; contribute to the update of the terminology on disaster risk reduction; use post-disaster reviews as opportunities to enhance learning and public policy and disseminate studies.^1^^,^^4^

Over the next 15 years, the framework aims to achieve the substantial reduction of disaster risk and losses in lives, livelihoods and health and in the economic, physical, social, cultural and environmental assets of persons, businesses, communities and countries. Voluntary commitments with a specific public health focus that have been agreed include: enhancing the resilience of national health systems through training and capacity development; strengthening the design and implementation of inclusive policies and social safety-net mechanisms, including access to basic health care services towards the eradication of poverty; finding durable solutions in the post-disaster phase to empower and assist people disproportionately affected by disasters, including those with life threatening and chronic disease; enhancing cooperation between health authorities and other relevant stakeholders to strengthen country capacity for disaster risk management for health; the implementation of the *International health regulations* *(2005)*^7^ and the building of resilient health systems; improving the resilience of new and existing critical infrastructure, including hospitals, to ensure that they remain safe, effective and operational during and after disasters, to provide live-saving and essential services; establishing a mechanism of case registry and a database of mortality caused by disaster to improve the prevention of morbidity and mortality and enhancing recovery schemes to provide psychosocial support and mental health services for all people in need.

The framework requires coordinated action across local, national, regional and international levels. Synergies across disaster risk reduction, the sustainable development goals and climate change policy need to be better recognized. This could considerably enhance management of disaster risks through capacity development and joint policy initiatives between the health sector and other sectors.^8^
